# Mild intermittent hypoxia may improve autonomic dysfunction in persons living with spinal cord injury: a preliminary snapshot

**DOI:** 10.3389/fnins.2025.1600772

**Published:** 2025-07-22

**Authors:** Alexandra E. Soltesz, Fei Zhao, Jill M. Wecht, Jason H. Mateika, Gino S. Panza

**Affiliations:** ^1^John D. Dingell Veterans Affairs Medical Center, Detroit, MI, United States; ^2^Translational Neuroscience Program, Wayne State University School of Medicine, Detroit, MI, United States; ^3^Division of Pulmonary, Critical Care and Sleep Medicine, Wayne State University School of Medicine, Detroit, MI, United States; ^4^Department of Spinal Cord Injury Research, James J. Peters Veterans Affairs Medical Center, Bronx, NY, United States; ^5^Department of Rehabilitation Medicine and Human Performance, Icahn School of Medicine at Mount Sinai, New York, NY, United States; ^6^Department of Physiology, Wayne State University, Detroit, MI, United States; ^7^Department of Internal Medicine, Wayne State University, Detroit, MI, United States; ^8^Department of Health Care Sciences, Program of Occupational Therapy, Wayne State University, Detroit, MI, United States

**Keywords:** tetraplegia, paraplegia, autonomic dysfunction, autonomic dysreflexia, orthostatic hypotension, mild intermittent hypoxia

## Abstract

Persons with spinal cord injuries often suffer from autonomic dysfunction, sleep disordered breathing, and impaired mitochondrial capacity. Current treatment options for these individuals are limited and often have significant side effects. Thus, new interventions that target multiple physiological systems and circumvent physical limitations would be a significant development for persons with spinal cord injury (pwSCI). One potential intervention is daily mild intermittent hypoxia (MIH) which has been shown to improve blood pressure control and upper airway function during sleep. Four individuals with chronic motor incomplete SCI underwent 8 days of MIH (ClinicalTrials.Gov ID #NCT05351827, https://clinicaltrials.gov/study/NCT05351827). The MIH protocol was administered each morning during wakefulness with end-tidal oxygen maintained at 55–60 mmHg. End-tidal carbon dioxide was maintained at + 3 mmHg above baseline during the MIH. Autonomic dysfunction (autonomic dysreflexia and orthostatic hypotension), sleep quality, upper airway function, mitochondrial capacity, and microvascular function were tested before, the day after, and 2 weeks following the MIH protocol. Systolic autonomic dysreflexia improved by 46% ± 14% and orthostatic hypotension improved by 160% ± 63% after MIH. Reductions in the apnea hypopnea index were observed, alongside a concurrent reduction in arousals during sleep. Upper airway function improved and mitochondrial capacity increased following 8 days of MIH. These preliminary data from four participants in an ongoing clinical trial suggest that 8 days of MIH may improve autonomic dysfunction, sleep quality, and mitochondrial capacity in pwSCI. The recruitment of additional participants is required to support these preliminary findings.

**Clinical trial registration:**

https://clinicaltrials.gov/study/NCT05351827, identifier NCT05351827.

## 1 Introduction

There are approximately 305,000 persons living with spinal cord injury (pwSCI) in the United States, with approximately 18,000 new cases each year (National SCI Statistical Center, 2020). The diverse sequela following SCI include, but are not limited to autonomic and cardiovascular dysfunction (Wecht et al., 2020; Ji et al., 2024), altered breathing function (including increased rates of sleep disordered breathing and sleep apnea) (Schilero et al., 2018; Graco et al., 2021), and reduced quality of life (Barker et al., 2009). These impairments following SCI are also concurrent with reductions in physical capacity and deconditioning leading to impaired mitochondrial capacity and metabolic dysfunction (Erickson et al., 2013; Nash et al., 2016), leading to an inactive lifestyle.

Spinal cord injury, and the secondary impairments associated with this injury, often leads to two distinct blood pressure (BP) responses referred to as autonomic dysreflexia (AD) and orthostatic hypotension (OH). AD is defined by a sudden rise in systolic BP equal to or greater than 20 mmHg. In addition, orthostatic hypotension (OH) is defined by a drop in systolic and diastolic BP that is equal to or greater than 20 and 10 mmHg, respectively. The sudden changes in BP reflect altered autonomic control which impedes activities of daily living and participation in rehabilitation, lengthens hospital stays, and increases stroke risk and cognitive dysfunction in pwSCI (Ji et al., 2024).

In addition to the direct impact of SCI on AD and OH, accompanying co-morbidities may also exacerbate AD and OH. Individuals living with SCI have a higher prevalence of sleep apnea compared to the general population (Graco et al., 2021). Hallmarks of sleep apnea include exposure to severe intermittent hypoxia and significant sleep fragmentation. Both hallmarks have been linked to increases in sympathetic activity and hypertension in some patients with intact spinal cords. Thus, it is possible that the presence of sleep apnea in pwSCI could contribute to exacerbating the autonomic and cardiovascular dysfunction associated with AD and OH.

Given the risks associated with AD and OH, the discovery of non-pharmacological and/or pharmacological interventions to mitigate or eliminate these autonomic impairments is important, since the effectiveness of existing non-pharmacological and/or pharmacological treatments is minimal (see discussion for details related to non-pharmacological and/or pharmacological treatments). Moreover, no studies to date have directly examined if improvement in sleep disordered breathing in individuals with SCI mitigates AD and OH. Mild intermittent hypoxia (MIH) is a novel intervention that contributes to the recovery of breathing in spinal cord injured animals and humans and may also have an impact on autonomic control of BP (Puri et al., 2021; Panza et al., 2022a). Recently, we showed that 15 days of MIH reduced blood pressure and sympathetic nervous system activity in individuals with sleep apnea and hypertension at rest (Panza et al., 2022a), and during sympathetic stimulation using hypoxia (Panza et al., 2022b). Thus, MIH might serve to directly impact autonomic and cardiovascular function, which could impact AD and OH in pwSCI. In addition, we have also shown that MIH leads to sustained increases in upper airway muscle activity along with increased upper airway patency (Panza et al., 2022a). In conjunction with changes in upper airway patency initiated by daily MIH, we have also reported that MIH may increase the arousal threshold (Alex et al., 2019) which may serve to improve sleep continuity. The improvements in upper airway function could reduce the severity of sleep apnea and consequently mitigate the autonomic dysfunction linked to AD and OH.

In association with the improvement in AD and OH, administering daily MIH might also improve microvascular function and mitochondrial capacity based on previous studies in humans with intact spinal cords (Shatilo et al., 2008; Serebrovska et al., 2019). If that is the case, this improvement could lead to increases in physical capacity and mitigate the physical deconditioning often associated with individuals living with SCI. Thus, the ongoing clinical trial that we are completing is designed to determine if daily exposure to MIH mitigates AD and OH either directly or via reductions in the severity of sleep disordered breathing. In addition, as a secondary outcome we are exploring whether this novel intervention leads to improved microvascular function and mitochondrial capacity.

## 2 Materials and methods

### 2.1 Ethical Approval

The institutional review board of Wayne State University and the John D. Dingell Veterans Affairs Medical Center approved this protocol (#IRB-22-04-4550) and this project is a registered clinical trial (ClinicalTrials.Gov ID #NCT05351827). Participants provided written informed consent prior to enrolling in the study.

### 2.2 Participants

The full study is a 2 arm, double blind, sham-controlled parallel group design. Participants with chronic motor incomplete SCI and signs or symptoms of autonomic dysfunction were recruited for the study. At the initial screening, two forms of evaluation [International Standards to Document Autonomic Function following SCI (ISAFSCI) and the Autonomic Dysfunction Following SCI (ADFSCI)] were used to document autonomic dysfunction. Autonomic dysfunction was determined by the response to question 16 and 22 on the ADFSCI and a score of 1 associated with any parameter of the ISAFSCI. Additionally, in response to a provocation, individuals had at least one systolic or diastolic BP measurement meet the 20/10 mmHg criteria associated with AD and OH to remain in the study following baseline testing. Although no official criteria for diastolic AD exists (Krassioukov et al., 2021), we elected to implement the criteria used to diagnose OH. Individuals with diabetes mellitus, congestive heart failure, or chronic obstructive pulmonary disease were excluded.

### 2.3 Mild intermittent hypoxia

Participants received 8 days of MIH completed within 2 weeks. During the administration of MIH, participants were fitted with a facemask (Hans Rudolph Inc., 7450 Series Oro-Nasal Mask) connected to a pneumotachometer (Hans Rudolph Inc., 4813 Series) with sample ports for oxygen and carbon dioxide (Hans Rudolph Inc., SmartLab™). The pneumotachometer was connected to a two-way non-rebreathing valve (Hans Rudolph Inc., Series 2600) which was connected to a 5-way stopcock (Hans Rudolph Inc., Series 2400). One valve of the 5-way stopcock was connected to a non-diffusible bag (Hans Rudolph Inc., Series 6100 Non-diffusing Gas Collection Bag) containing 8% oxygen, and the other port was connected to another non-diffusible bag containing 100% oxygen. End-tidal gases were manually controlled using a gas mixer (Aalborg Instruments, Model PX Multi-Flow Tube Meters) to maintain the flow of 100% oxygen and carbon dioxide into the inspiratory line. Participants were fitted with a pulse oximeter (Nonin Medical Inc., 8000AA Series Reusable Finger Clip Pulse Oximetry Sensor), 3-lead ECG (Norav Medical Inc., ECGUSB1D-EX), and a beat-to-beat BP system (CNSystems Medizintechnik GmbH, CNAP^®^ Monitor 500).

**FIGURE 1:**
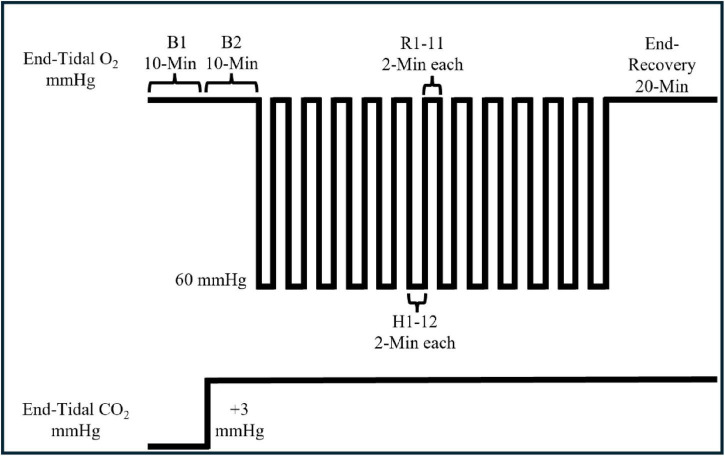
Schematic of the mild intermittent hypoxia protocol. Tracings illustrates the changes in partial pressure of end-tidal oxygen (P_*ET*_O_2_) (upper) and carbon dioxide (P_*ET*_CO_2_) (lower). This protocol was repeated for 8 days over 2 weeks. There was no difference in baseline P_*ET*_CO_2_ on day 1 versus day 8 (38.22 ± 5.06 vs 39.62 ± 7.60). Also, the average increase in P_*ET*_CO_2_ throughout the protocol on day 1 and day 8 were similar (+3.99 ± 0.93 and +2.96 ± 0.56). Likewise, there was no difference in baseline P_*ET*_O_2_ on day 1 versus day 8 (103.15 ± 7.40 vs 103.34 ± 6.13) with the change in P_*ET*_O_2_ during hypoxia also showing no difference (–44.73 ± 5.5 vs –48.39 ± 2.0). The average P_*ET*_O_2_ administered during the hypoxic episodes on day 1 vs. day 8 is 58.34 ± 2.81 vs 56.10 ± 2.22 mmHg.

The MIH protocol began with participants breathing room air for 10 min without the two-way non-rebreathing valve connected (i.e., eupneic breathing, B1) to establish baseline ventilation and end-tidal gases. After the conclusion of the 10 min, the two-way non-rebreathing valve was connected to the pneumotachometer. Thereafter, a 10-min hypercapnic baseline (B2) was established, during which end-tidal carbon dioxide (CO_2_) was maintained +3 mmHg above the CO_2_ level established during B1. The CO_2_ level established during B2 was maintained throughout the remainder of the protocol. During the 2-min hypoxic bouts, participants inspired 8% oxygen from the non-diffusible bag with 100% oxygen titrated into the inspiratory line to maintain end-tidal oxygen between 55–60 mmHg. At the conclusion of each hypoxic bout, individuals were given 1 breath of 100% oxygen, after which participants breathed room air titrated with the additional CO_2_ for 2 min. This protocol was repeated 12 times and following the last hypoxic bout individuals breathed room air for 20 min. The total hypoxic protocol was completed in 86 min. See Figure 1.

### 2.4 Autonomic dysfunction: autonomic dysreflexia and orthostatic hypotension

Blood pressure responses to AD and OH provocations were tested before, the morning after, and 2 weeks following the 8-day MIH protocol. During all autonomic testing, participants were fit with an ECG and finger BP monitor. To initiate AD, participants were fitted with an occlusion cuff (Delfi Medical Innovations Inc.) on the upper thigh. After 5 min of uninterrupted BP monitoring, the thigh cuff was inflated to 300 mmHg for a duration of 6 min. The magnitude of AD was calculated as the difference between the maximum SBP and DBP reached during the 6-min of occlusion and the average BP measured during the final 2 min of baseline prior to occlusion. Blood pressure peaks associated with spasms, talking, or movement, were excluded from analysis.

**FIGURE 2:**
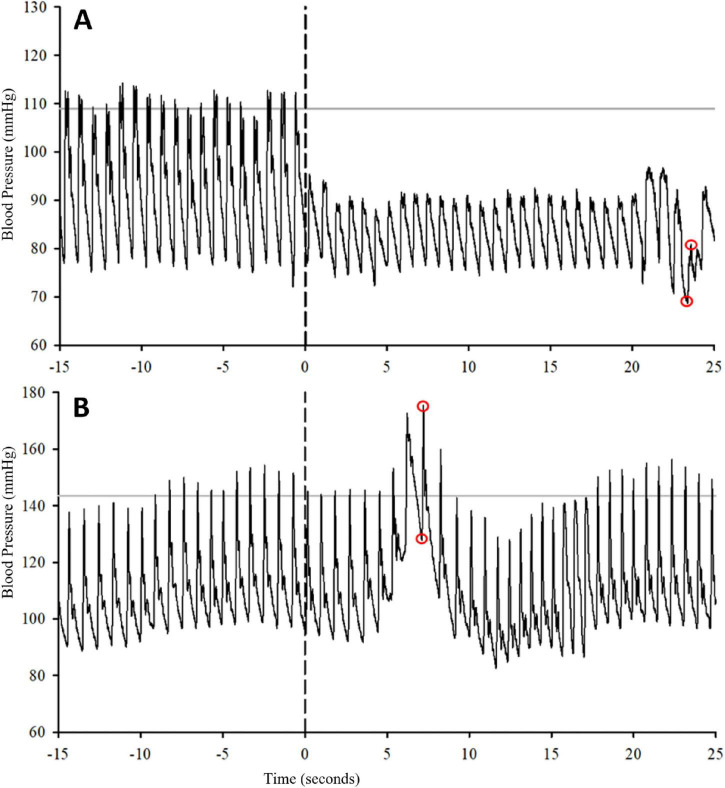
Representative traces for OH **(A)** and AD **(B)** during initial autonomic testing. Gray horizontal lines represent 2–5-min averages of baseline blood pressure. Vertical dashed lines represent the start of the provocation. For OH, the provocation was the sit-up test. For AD, the provocation is the 300-mmHg occlusion torniquet around the upper thigh. The red circles represent the data points that were selected for the window of time shown and may not represent the data selected for the group means. The initial 15 s is shown for clarity. For OH, the lowest systolic and diastolic BP was selected. For AD, the peak systolic and diastolic BP during the 6-min occlusion test was selected. The difference in these values from resting baseline was used as the magnitude of OH and AD, respectively. Any data points associated with a spasm, talking, or movement were excluded from analysis. Data points were only selected if they had a corresponding R-wave from the EKG.

To test for OH, a modified sit-up test was used. Participants laid supine for 5 minutes on a stretcher. Individuals were then moved to approximately 100° at the hip with their legs extended horizontally, in approximately 1–2 s. During the postural change, the hand with the beat-to-beat BP device was held in place over the heart using a standard arm-sling. The difference between the lowest SBP and DBP within 3 min of the position change (Krassioukov et al., 2021) and the average of the preceding 2 min of baseline BP was used to calculate the magnitude of OH. All BP values were only selected if they were immediately preceded by an R-wave, and were not concurrent with spasms, talking, or other non-rest wakefulness breathing actions. Representative responses to these tests are presented in Figure 2.

### 2.5 Sleep quality and upper airway function

A split-night polysomnography (PSG, Nox Medical USA, Nox A1s PSG System) was conducted before, the night after, and 2 weeks after the MIH protocol. The split-night PSG consisted of a baseline PSG (initial 3–4 h) to determine the presence and severity of sleep apnea, and overall sleep quality. If individuals had greater than 5 events per hour, they were switched to a continuous positive airway pressure (CPAP) titration protocol to determine the therapeutic pressure (TP) and upper airway critical closing pressure (CCP). Sleep studies were manually staged and scored according to the American Academy of Sleep Medicine Manual (Version 3) using Noxturnal software (Nox Medical USA, Noxturnal Software System 6.0). All measures were taken during non-rapid eye movement (NREM) sleep, and sleep stages are represented as a percentage of total sleep time.

Polysomnograms were conducted using electroencephalography (F1/M2, F2/M1, C1/M2, C2/M1, O1/M2, O2/M1 arrangement). Participants wore a mask and pneumotachometer to record airflow. The titration study was completed using a CPAP machine (Resmed, AirSense 11) including a pneumotachometer (Hans Rudolph Inc., 3700 Series). To determine TP, the CPAP was increased by 1 cmH_2_O every 10 min until minimum airway obstruction was visible in the flow signal and sleep was uninterrupted. The pressure was increased until no airflow limitation was noticed, or the pressure resulted in hypocapnia or disturbed sleep. The pressure associated with hypocapnia or disturbed sleep was considered to be 1 cmH_2_O above TP and the previous pressure was determined to be the TP (Panza et al., 2022a). After the TP was determined, participants underwent a protocol to evaluate airway collapsibility, during which the positive airway pressure was decreased by 1 cmH_2_O every 5 min until an apnea, was observed (CCP). The ResMed device does not provide CPAP below 4 cmH_2_O, thus in participants with no obstructive apnea during the step-down protocol, average maximum inspiratory flow was plotted against CPAP and fit to a linear or quadratic equation. The best fit equation was used to determine the pressure at which inspiratory flow was predicted to be zero, and this pressure was used as the CCP (Panza et al., 2022a). Participants with sleep apnea were treated with a CPAP only for the duration of the 8 days of MIH and the CPAP machine was returned after post-MIH testing.

### 2.6 Mitochondrial and microvascular function

To determine mitochondrial and microvascular function, a near infrared spectroscopy (NIRS) device (Moxy, Moxy Sensor) was placed over the medial gastrocnemius of the occluded limb during the AD test. Baseline oxyhemoglobin, deoxyhemoglobin and tissue saturation index were recorded at rest, throughout the occlusion, and for 6 min during reperfusion (i.e., release of the thigh occlusion cuff). Tissue saturation index (TSI) was the signal used to determine mitochondrial capacity and microvascular function. Resting TSI was averaged for 60 s prior to occlusion. The TSI waveform was then normalized to this baseline throughout occlusion and reperfusion. Data collected during the final 20 s of occlusion was averaged to determine the maximum percentage of oxygen consumed and this value was used to quantify mitochondrial capacity. For example, if the minimum percentage of oxygen during the final 20 s of occlusion was 20%, then the mitochondrial capacity was determined to be 80%. The time constant (i.e., tau - the time required to achieve 63% of the maximum hyperemic response) associated with reperfusion was used as an indicator of microvascular function. To account for changes in amplitude, which elongate Tau, the Tau measured during hyperemia was normalized to the amplitude of reoxygenation (minimum achieved during occlusion, to maximum achieved during reperfusion).

## 3 Results

### 3.1 Statistical analysis

As this is preliminary data of an ongoing trial, no statistical comparisons are made. The data presented here are from the first four experimental (i.e., MIH group) participants in which blinded analysis was completed, and only descriptive data is provided across time points. Participant characteristics are presented in Table 1. All data are presented as mean ± standard deviation.

**TABLE 1:** Participant characteristics.

Participant	Age	Height (cm)	Weight (kg)	BMI	Supine SBP	Supine DBP	AHI	Injury level	AIS	Race
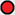 1-M	49.00	167.00	57.24	20.50	101.00	71.00	68.00	C4	D	Black
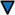 2-F	37.00	180.00	72.00	22.20	108.00	71.00	10.20	T1	C	Black
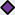 3-M	46.00	183.00	105.00	31.40	136.00	86.00	35.70	C6	C	Black
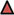 4-M	44.00	152.00	54.00	23.40	107.00	78.00	28.80	C4/C7	C	Asian
Mean	44.00	170.50	72.06	24.38	113.00	76.50	35.68			
Standard deviation	5.10	14.15	23.31	4.83	15.64	7.14	24.09			

### 3.2 Orthostatic hypotension

**FIGURE 3:**
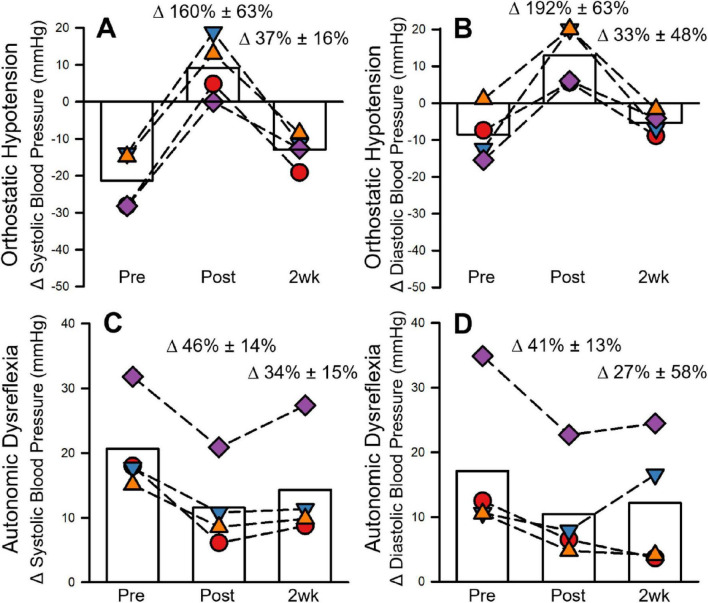
Autonomic dysfunction before, after, and 2 weeks following 8 days of mild intermittent hypoxia (MIH). Individual data points are represented with scatter plots. Group means are represented by the open bar graphs. Group means are presented in order of testing (i.e., pre, post, 2-weeks following MIH). The magnitude of systolic OH is represented in panel **(A)** and diastolic OH is represented in panel **(B)**. The magnitude of systolic AD is presented in panel **(C)** and diastolic AD in panel **(D)**. Δ = change. Please note, each participant is represented by the same symbol across all graphs. Standard deviations are not presented since individual data are presented in each graph.

All four participants met the criteria for either systolic or diastolic OH or AD during baseline testing. Three of the four participants met the criteria for systolic or diastolic OH. The average decrease in systolic BP during OH was −21.26 ± 7.99 mmHg. The decrease in BP during systolic OH was eliminated after MIH (9.16 ± 8.40 mmHg; 160 ± 63% change from baseline) and reduced 2 weeks (−12.89 ± 4.51 mmHg; 37% ± 16% change from baseline) following MIH. Diastolic OH was evident in two participants prior to MIH, despite the absence of systolic OH. Prior to MIH the decrease in BP during diastolic OH was −8.52 ± 7.22 mmHg. In contrast, the change in BP 1 day and 2 weeks after MIH was 12.94 ± 8.23 mmHg (192% ± 63% change from baseline) and −5.38 ± 3.18 mmHg (33% ± 48% change from baseline), respectively (See Figures 3A, B). Diastolic OH was not evident at baseline in one participant, however despite this absence, this participant’s diastolic BP during OH increased resulting in a 1716% improvement after MIH. This outlier was excluded from the average calculation of diastolic OH. Heart rate increased during OH at baseline (14.19 ± 9.71 beats/minute), 1 day (20.44 ± 14.11 beats/minute), and 2-weeks after MIH (18.18 ± 13.46 beats/minute).

### 3.3 Autonomic dysreflexia

Only one participant had systolic AD, but all four participants reached inclusion criteria for diastolic AD. The average increase in SBP during AD was 20.67 ± 7.55 mmHg prior to MIH. The increase was smaller 1 day (11.60 ± 6.50 mmHg; −46% ± 14% of baseline) and 2 weeks (14.34 ± 8.77 mmHg; 34% ± 15% of baseline) following MIH. The increase in DBP during AD was 17.15 ± 11.88 mmHg prior to MIH. The increase in DBP during AD was reduced 1 day (10.48 ± 8.26 mmHg; 41% ± 13% compared to baseline) and 2 weeks (12.20 ± 5.07 mmHg; 15% ± 33% compared to baseline) following MIH (See Figures 3C, D). Heart rate increased during AD at baseline (9.03 ± 9.74 beats/minute) and there was no clear effect of MIH observed 1 day (12.50 ± 10.16 beats/minute) and 2-weeks (9.57 ± 5.13 beats/minute) following MIH.

### 3.4 Mitochondrial and microvascular function

**FIGURE 4:**
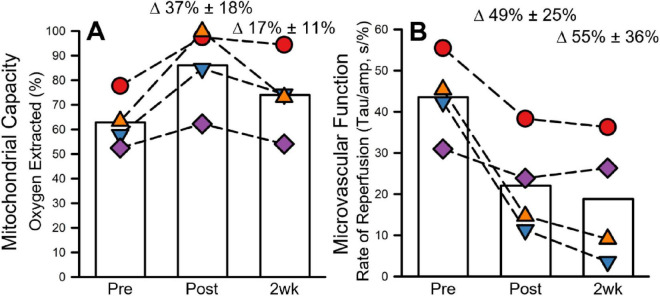
Mitochondrial and microvascular function before, after, and 2 weeks following 8 days of mild intermittent hypoxia (MIH). Figure **(A)** presents mitochondrial capacity as measured by the amount of oxygen extracted during the occlusion test. Figure **(B)** presents microvascular function as measured by the Tau normalized to the amplitude change (See Section “2.6 Mitochondrial and microvascular function” for mathematical computation and justification). All figures present individuals (scatter) and group means (open bars) before, after and 2-weeks following the 8-day MIH protocol.

Prior to MIH, mitochondrial capacity was 62.83% ± 10.85%. Mitochondrial capacity increased to 86.04% ± 17.19% (37% ± 18% compared to baseline) and 73.97% ± 16.48% (17% ± 11% compared to baseline) 1 day and 2 weeks following MIH, respectively (Figure 4A). Prior to MIH, the time constant associated with microvascular reperfusion was 43.54 ± 10.09 s/%. The time constant was reduced 1 day (22.03 ± 12.09 s/%; 49% ± 25% compared to baseline) and 2-weeks (18.81 ± 15.13 s/%; 55% ± 36% compared to baseline) following MIH (Figure 4B).

### 3.5 Sleep

The percentage of N1 sleep (light sleep) was 51.60% ± 10.89% prior to MIH. This percentage decreased to 32.95% ± 11.52% and 23.35% ± 15.76% 1 day and 2 weeks following MIH, respectively. The percentage of N3 sleep was 2.53% ± 2.88% prior to MIH and increased to 10.43% ± 10.87% 1 day and 13.48% ± 14.12% 2 weeks following MIH, respectively. REM sleep was not impacted by MIH (Baseline vs. 1 day vs. 2 weeks; 3.00% ± 6.00% vs. 3.55% ± 7.10% vs. 3.30% ± 6.40%).

**FIGURE 5:**
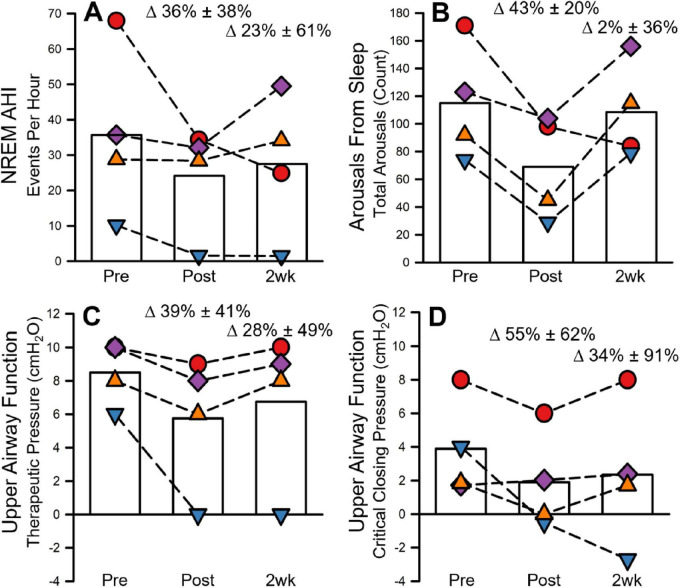
Sleep and upper airway function before, after, and 2 weeks following 8 days of mild intermittent hypoxia (MIH). Figure **(A)** presents sleep apnea severity (NREM AHI) in events per hour. Figure **(B)** shows the change in arousal number during the initial 3–4 h of sleep of the PSG study. Figures **(C)** and **(D)** represent upper airway function during the titration portion of the split-night sleep study. Figure **(C)** shows the therapeutic pressure required to treat sleep apnea. Figure **(D)** presents upper airway collapsibility as characterized by the critical closing pressure. All figures present individual (scatter) and group means (open bars) before, after and 2-weeks following the 8-day MIH protocol. NREM, non-rapid eye movement sleep; AHI, apnea hypopnea index (events/hour).

Sleep apnea (AHI = 35.68 ± 24.09 events/hour) was evident in all participants prior to MIH. Consequently, all participants were treated nightly with CPAP. After MIH, the AHI was reduced to 24.13 ± 15.22 events/hour (36% ± 38% reduction compared to baseline) and 27.50 ± 20.09 events/hour (23% ± 61% reduction compared to baseline) 1 day and 2 weeks following MIH, respectively (See Figure 5A).

The average total number of arousals during sleep was 115.00 ± 42.47 prior to MIH. The number of arousals decreased (69.00 ± 37.60; 43% ± 20% reduction compared to baseline) 1 day following MIH and returned to baseline values (108.50 ± 35.44) 2 weeks following MIH (See Figure 5B). In line with the changes in AHI, the number of respiratory arousals (85.50 ± 56.56) during sleep, prior to MIH, decreased 1 day (49.00 ± 28.32) and 2 weeks (72.50 ± 59.91) following MIH. The number of spontaneous arousals during sleep prior to MIH (29.00 ± 15.94) decreased 1 day after MIH (20.00 ± 11.49) and returned to baseline levels 2 weeks following MIH (36.00 ± 27.56).

All four participants were naïve to CPAP prior to participating in the study. The TP measured during sleep prior to MIH (8.50 ± 1.91 cmH_2_O) was decreased 1 day (5.75 ± 4.03 cmH_2_O; 39% ± 41% decrease compared to baseline) and 2 weeks after MIH (6.75 ± 4.57 cmH_2_O; 28% ± 49% decrease compared to baseline). Similarly, the critical closing pressure measured during sleep prior to MIH (3.90 ± 2.93 cmH_2_O) was reduced 1 day (1.89 ± 2.94 cmH_2_O; 55% ± 62% compared to baseline) and 2 weeks (2.35 ± 4.39 cmH_2_O; 34% ± 91% decrease compared to baseline) following MIH (See Figures 5C, D).

## 4 Discussion

The preliminary findings from this trial support our hypothesis that 8 days of MIH has the potential to improve AD and OH in pwSCI. Additionally, these data suggest that MIH may also improve mitochondrial and microvascular function. Moreover, these preliminary data provide initial support for improvements in sleep quality and upper airway function in pwSCI. However, based on the current sample size, we did not attempt to correlate these outcomes.

### 4.1 Orthostatic Hypotension and autonomic dysreflexia

Interventions targeting autonomic control of BP in pwSCI are scant despite the known importance, and impact, of altered BP control in this group (Krassioukov et al., 2021). Various non-pharmaceutical and pharmaceutical treatments have been used with limited success (Wecht and Bauman, 2018). Mitigating extravascular blood volume shifts with salt and water intake does not alter OH, although it does improve cerebrovascular function by reducing the symptoms of orthostatic intolerance (Claydon and Hainsworth, 2004). However, a clinical trial has not been designed to provide further support for this latter finding (Wecht and Bauman, 2018). Likewise, the efficacy of pressure garments has been investigated, but evidence supporting improvements in OH remains mixed (Huang et al., 1983; Hopman et al., 1998). The effectiveness of pharmaceutical treatments for OH is equally unclear. The drug used most often for the treatment of OH is midodrine, a selective alpha-agonist that increases vascular tone (Wecht and Bauman, 2018). However, a recent study showed that despite the improvement of resting blood pressure in individuals living with OH, symptoms of AD worsened following the administration of midodrine (Katzelnick et al., 2019; Wecht et al., 2023). In our data, the magnitude change in systolic OH seems to be similar between those above and below the Δ 20 mmHg criteria potentially suggesting a similar responsiveness to daily MIH despite potential baseline differences in systolic OH. Likewise, we saw similar responses in diastolic OH. Of mention, one participant (orange triangle) did not have systolic or diastolic OH, but had similar changes following MIH. One participant (blue triangle) did not have systolic OH, but did have diastolic OH, and this individual had the largest change in diastolic OH out of the four participants. This could suggest that this participant had more spared sympathetic neurons that may have been augmented with daily MIH.

Preventative treatment for AD is far less studied than OH. Prophylactic treatment of AD includes avoiding situations that provoke the condition, which include a full bladder or bowel, tight socks, belt or pants, or sitting for a prolonged period of time (Krassioukov et al., 2021). Unfortunately, pwSCI often do not respond to the sensations associated with a full bladder or bowel, or tight clothing. Likewise, some pwSCI cannot move to change positions independently. Consequently, rapid, uncontrolled, and severe elevations in BP can occur up to 41 times/day (Hubli et al., 2015), which are often asymptomatic (Kirshblum et al., 2002) and can be fatal (Wan and Krassioukov, 2014). Despite the known increased risk of stroke (Wu et al., 2012) and potential life threatening increases in BP (Wan and Krassioukov, 2014), the development of new preventative strategies are scant as treatment options remain focused on mitigating acute bouts of AD (Krassioukov et al., 2021). The primary modality used to mitigate acute bouts of AD are anti-hypertensive drugs. A seminal study in this area reported a significant reduction in the magnitude of AD during penile stimulation when pwSCI were given Prazosin, a selective alpha adrenergic blocker (Phillips et al., 2015). Although this finding supports the use of alpha adrenergic blockers to minimize blood pressure increases during AD provoking situations (i.e., sperm retrieval), the use of alpha adrenergic blockers as a preventative treatment is not recommended (Krassioukov et al., 2021). Thus, as it pertains to preventative interventions, pharmaceutical management of OH and AD may exacerbate BP outcomes further supporting the importance of developing new strategies to improve BP control in pwSCI. Unlike pharmaceutical treatment, our intervention currently shows improvements in both AD and OH. Of note for AD, the largest magnitude of AD was found in the participant who was hypertensive at rest (purple diamond) which may suggests more spared sympathetic neurons. Interestingly, this participant appears to have the same magnitude reduction as the other participants, and potentially a larger reduction in diastolic AD. These findings are exciting considering MIH is associated with sympathoexcitation, but daily exposure shows improvement to provocation, as opposed to resting BP. Ultimately, this suggests there are other mechanisms influencing BP regulation in these participants.

Mild intermittent hypoxia is a perturbation often used to study the control of breathing as well as the pathophysiological outcomes associated with sleep apnea (Puri et al., 2021; Badran and Gozal, 2025). More recently, daily exposure to MIH has been used to initiate beneficial cardiovascular and motor outcomes in humans (Puri et al., 2021; Vose et al., 2022). Improvements in BP following exposure to MIH have been reported in individuals with myocardial infarction (Burtscher et al., 2004), pre-diabetes and hypertension (Serebrovska et al., 2017; Panza et al., 2022a), as well as older individuals with mild cognitive impairment (Wang et al., 2020). In addition to initiating reductions in blood pressure in hypertensive individuals, we also reported that treatment with MIH leads to a reduction in the BP response to hypoxia (Panza et al., 2022b). This finding suggests that the BP response to a similar sympathetic stimulus is blunted following treatment with MIH. Based on this finding it may be postulated that lowering resting BP and BP responses to a sympathetic perturbation may worsen OH, however our data suggests the opposite. OH might be improved because of increases in muscle sympathetic nerve activity following MIH. This may or may not be concurrent with changes in BP thorough review can be found here [(Puri et al., 2021)], but this mechanism would worsen AD. Thus, other mechanisms that influence BP control in pwSCI could be responsible for the improvements in OH and AD that we observed. The potential mechanisms include changes in mitochondrial and local microvascular function.

### 4.2 Mitochondrial and microvascular function

Our study suggests that daily exposure to MIH may improve mitochondrial and microvascular function. Currently, we are unaware of any evidence directly investigating mitochondrial capacity following daily MIH. Likewise, comparing vascular function following daily exposure to MIH is difficult due to the variability in MIH protocols (Puri et al., 2021; Panza et al., 2023a). There is one study in untrained senior men which showed that daily exposure to MIH improved microvascular and endothelial function (Shatilo et al., 2008). The potential mechanism for improving mitochondrial function is likely upregulation of reactive oxygen species. It is known that during hypoxia there is an upregulation of reactive oxygen species, and these impact intracellular calcium signaling (Nanduri et al., 2013) which activate peroxisome proliferator-activated receptor-gamma coactivator 1-alpha (PGC1α). The activation of PGC1α leads to increased mitochondrial biogenesis and Kreb cycle enzymes (Mallet et al., 2018). Furthermore, MIH is associated with upregulation of pyruvate dehydrogenase kinase (Serebrovska et al., 2019) further supporting improvements in bioenergetic pathways associated with mitochondrial function. Finally, other studies have linked improvements in BP control to improved nitric oxide synthesis (Lyamina et al., 2011; Muangritdech et al., 2020), which is known to impact microvascular function (Durán et al., 2010) and mitochondrial function (Erusalimsky and Moncada, 2007). Ultimately, these peripheral mechanisms help explain improvements in metabolic function in neurologically intact humans following MIH (Serebrovskaya et al., 2008; Serebrovska et al., 2017; Serebrovska et al., 2019) that may be implicated in BP control. For example, one participant (purple diamond) was hypertensive at rest and had the largest magnitude of AD. This participant also had the lowest mitochondrial capacity of the 4 individuals. Although we suggest mitochondrial capacity is an important physiological function for BP control, it cannot solely explain the changes in AD found here as this participant did not have the same magnitude of improvement in mitochondrial capacity as the other participants. Thus, improvements in mitochondrial and microvascular function may play an important role in mitigating the rise in BP during sympathetic activation.

### 4.3 Sleep

Arguably the most studied phenomena following exposure to MIH, in humans, is the neural plasticity of breathing which is termed long-term facilitation (LTF). LTF of minute ventilation is characterized by a sustained increase in minute ventilation, compared to baseline, following exposure to MIH (Puri et al., 2021). Unique to humans, as opposed to phrenic LTF in animals, LTF of minute ventilation requires the maintenance of end-tidal CO_2_ above baseline values (Harris et al., 2006). The magnitude of LTF is dependent on the total hypoxic burden associated with the protocol and the chemoreflex sensitivity to hypoxia (Panza et al., 2023b). Previous studies have also shown that exposure to MIH leads to LTF of upper airway muscle activity ultimately in addition to increased upper airway patency (NB as measured by the critical closing pressure) and a reduced therapeutic pressure to treat sleep apnea (Panza et al., 2022a) which explains the improvement in AHI following MIH (Zha et al., 2023). Thus, LTF of upper airway muscle activity might explain the reduction in the AHI that was observed in our participants. However, there are instances where experimental MIH, administered immediately before sleep, has shown to worsen AHI (Yokhana et al., 2012). The presumption is that the worsening of sleep apnea severity is due to increases in peripheral chemoreceptor sensitivity and loop gain (Alex et al., 2019). In our dataset, there appears to be two strong responders to disease severity (Figure 5A, red circle and blue triangle) as the other two individuals show minimal reductions in AHI. One individual had a resolution of sleep apnea, but her events were primarily obstructive hypopneas without severe airway limitation. Conversely, the other individual (red circle) had an obstructive apnea index of 49.4 that reduced to 3.0 after MIH. For these individuals, we did not observe an increase in the peripheral chemoreceptor sensitivity on day 8 compared to day 1 of MIH.

Furthermore, we previously reported that MIH increases the arousal threshold, making it more difficult to awaken from sleep (Alex et al., 2019; Panza et al., 2019). An increase in the arousal threshold likely explains the reduction in the arousals observed in the present study. This potential increase in arousal threshold and observed decrease in arousal count may contribute to an improvement in sleep quality, as evidenced by the decrease in light (i.e., N1) sleep percentage and the associated increase in deep sleep (i.e., N3) sleep percentage. However, to fully interpret these data the completion of the sham group is required to account for the impact of CPAP during the 2-week protocol, and any night-to-night variation in these outcomes.

It is well known that impaired sleep alters autonomic function, but comprehensive findings on the interplay of BP control and impaired sleep are limited in pwSCI (Panza et al., 2021; Ji et al., 2024). However, there is evidence that suggests that treatment of impaired sleep with CPAP reduces symptomatology associated with autonomic dysfunction in pwSCI (Brown et al., 2018). We also know that adherence to CPAP improves microvascular function in those with sleep apnea (Bischof et al., 2020), further implicating mitochondrial and microvascular function in these data. Thus, the literature, in combination with these preliminary data, suggest that daily MIH may have a multifaceted impact on cardiovascular function and sleep quality in pwSCI.

To summarize, the currently available evidence suggests acute MIH augments sympathetic activity (Ahmadian et al., 2025a), which can stabilize resting BP in rats (Ahmadian et al., 2025b) but also may explain the improvements in OH in pwSCI. However, augmenting sympathetic activity with MIH would worsen AD. Conversely, daily exposure to MIH has been shown lower BP in hypertensive individuals (Panza et al., 2022a; Panza et al., 2022b) suggesting other mechanisms are involved in BP control (Burtscher et al., 2023). One potential mechanism that may influence BP control following daily MIH are changes in mitochondrial and microvascular function. It is possible that these mechanisms can blunt the rise in BP during sympathetic activation providing a plausible explanation on how AD may improve with daily MIH.

### 4.4 Limitations and future directions

The findings of this preliminary study should be interpreted with caution. Given the small sample size, our results are not generalizable, and completion of the clinical trial is necessary to confirm our results. Nevertheless, the current dataset is not highly variable when comparisons are made between baseline measures and measures obtained 1 day after treatment. However, there is an increase in variability at the 2-week time point, particularly in the sleep related outcomes. Independent of the present trends, individual variability may change as more participants are recruited into the trial.

The protocol used to elicit AD is another potential limitation. We were cautious in our approach to eliciting AD because the safety and efficacy associated with inducing AD following exposure to MIH is presently unknown. Thus, participants were placed in a seated position as a starting point. This body position could impact the generalizability of our results as they do not mimic the same position or severity of responses that one may experience in everyday life. For example, our protocol did not elicit a level of AD considered to be associated with a hypertensive crisis. On the other hand, we rationalized that occluding the upper thigh would initiate AD, based on stimuli known to elicit AD coupled with the assumption that the blood trapped in the lower limb following occlusion would reduce venous return and elicit a sympathetic response. Moreover, the stimulus to elicit AD was identical within and across participants. Indeed, despite the potential limitations, AD was induced in all participants using our protocol. If the findings from this study are confirmed following completion of the trial, then future studies could be designed to investigate the impact of MIH on more severe stimuli that elicit AD and OH.

Lastly, all four participants were treated with CPAP during the 8 days of exposure to MIH. Thus, the CPAP treatment could have influenced our results. However, we do not think this is the case, since improvements in blood pressure with CPAP typically take 6 months or longer (Fava et al., 2014; Lui et al., 2021). The impact of CPAP on these outcomes will be addressed with the inclusion of data from the sham group at the completion of the clinical trial. Finally, the completion of the sham group will improve our interpretation of the sleep outcomes since it remains possible that the participants became more comfortable with sleeping in the research laboratory and with the attached equipment, subsequently influencing their sleep related outcomes.

## 5 Conclusion

In conclusion, 8 days of MIH with sustained slight hypercapnia administered in the morning during wakefulness improved autonomic function, sleep quality, mitochondrial capacity, and microvascular function in individuals with motor incomplete SCI that have signs and symptoms of autonomic dysfunction. These findings suggest that MIH may be a non-motor or pharmaceutical interventional strategy to improve multiple physiological functions known to be impaired following SCI. If these data are confirmed after comparisons to sham participants are completed, MIH would be the first intervention to improve both AD and OH in pwSCI.

## Data Availability

The raw data supporting the conclusions of this article will be made available by the authors, without undue reservation.
